# Variation in genome size and karyotype among closely related aphid parasitoids (Hymenoptera, Aphelinidae)

**DOI:** 10.3897/CompCytogen.v11i1.10872

**Published:** 2017-02-23

**Authors:** Vladimir E. Gokhman, Kristen L. Kuhn, James B. Woolley, Keith R. Hopper

**Affiliations:** 1 Botanical Garden, Moscow State University, Moscow, Russia; 2 Beneficial Insects Introduction Research Unit, ARS-USDA, 501 South Chapel Street, Newark, Delaware, United States of America; 3 Department of Entomology, Texas A&M University, College Station, Texas, United States of America

**Keywords:** Aphelinidae, *Aphelinus*, parasitoid, genome size, flow cytometry, karyotype

## Abstract

Genome sizes were measured and determined for the karyotypes of nine species of aphid parasitoids in the genus *Aphelinus* Dalman,1820. Large differences in genome size and karyotype were found between *Aphelinus* species, which is surprising given the similarity in their morphology and life history. Genome sizes estimated from flow cytometry were larger for species in the *Aphelinus mali* (Haldeman, 1851) complex than those for the species in the *Aphelinus daucicola* Kurdjumov, 1913 and *Aphelinus varipes* (Förster,1841) complexes. Haploid karyotypes of the *Aphelinus daucicola* and *Aphelinus mali* complexes comprised five metacentric chromosomes of similar size, whereas those of the *Aphelinus varipes* complex had four chromosomes, including a larger and a smaller metacentric chromosome and two small acrocentric chromosomes or a large metacentric and three smaller acrocentric chromosomes. Total lengths of female haploid chromosome sets correlated with genome sizes estimated from flow cytometry. Phylogenetic analysis of karyotypic variation revealed a chromosomal fusion together with pericentric inversions in the common ancestor of the *Aphelinus varipes* complex and further pericentric inversions in the clade comprising *Aphelinus kurdjumovi* Mercet, 1930 and *Aphelinus hordei* Kurdjumov, 1913. Fluorescence *in situ* hybridization with a 28S ribosomal DNA probe revealed a single site on chromosomes of the haploid karyotype of *Aphelinus coreae* Hopper & Woolley, 2012. The differences in genome size and total chromosome length between species complexes matched the phylogenetic divergence between them.

## Introduction

Genome size estimates and karyotypic studies provide data for comparative research at various taxonomic levels and allow evaluation of phylogenetic associations (Gokhman 2009, Hanrahan and Johnston 2011, Lopes et al. 2009). The completeness of genome assemblies can be difficult to assess, and independent estimates of genome size can aid in the assessment of completeness of genome assemblies (Gregory et al. 2013). Flow cytometry and Feulgen densitometry have been used to accurately measure genome size, and both methods have been extensively validated and various sources of error have been minimized through best-practice protocols (Gregory et al. 2013, Hare and Johnston 2011). Karyotypes can further help in assessing genetic linkage maps, and thus aid in mapping quantitative trait loci (Gokhman and Kuznetsova 2006). To visualize karyotypic features, various techniques of conventional and differential staining of chromosomes have been used, including fluorescence *in situ* hybridization (FISH), which allows physical mapping of DNA sequences onto chromosomes (Gadau et al. 2014, Macgregor and Varley 1988). Increasing the numbers of genome size estimates and karyotypes across the tree of life provides resources for the advancement of evolutionary genomics (Jacobson et al. 2013, Sharakhova et al. 2014). Furthermore, both flow cytometry and karyotypes can be used to detect cryptic species (Baur et al. 2014, Vergilino et al. 2012).

Genome size estimates have been published for more than 13,000 species of animals and plants (Animal Genome Size Database, http://www.genomesize.com; Plant DNA C-values Database, http://data.kew.org/cvalues; accessed 29 August 2014). There are currently 930 estimates of insect genome size in the Animal Genome Size Database, 152 of which are for species of Hymenoptera, and these genome sizes range from 98 to 1115 Mb. Genome size is usually considered constant within species, and limited intraspecific variation is a standard assumption in measurement and comparison of genome sizes. However, genome size can vary widely between closely related species (Gregory and Johnston 2008) and even within species (Biemont 2008, Bosco et al. 2007). The most common source of inter- and intraspecific genome size variation is differing amounts of repetitive DNA (Biemont 2008, Bosco et al. 2007). Differences in chromosome size can result from differences in heterochromatin content and amount of repetitive DNA in euchromatin, and differences in both chromosome size and number can result from fissions and fusions (Gokhman 2009, White 1973). Chromosome numbers and other karyotypic features have been published for about 70,000 species of plants and animals (Rice et al. 2014, White 1973; Tree of Sex: A database of sexual systems, doi: 10.1038/sdata.2014.15), including more than 1,500 species of Hymenoptera, whose haploid chromosome numbers range from 1 to 60 (Gokhman 2009, Ross et al. 2015).

Here we report genome size estimates and karyotypes for males and females in nine species of *Aphelinus* Dalman, 1820 (Hymenoptera: Chalcidoidea: Aphelinidae) all of which are parasitoids of aphids. Parasitoids are free-living as adults, but are parasitic as larvae, and represent one of the most species-rich groups of insects, constituting more than 10% of all described insect species (Eggleton and Belshaw 1992, Heraty et al. 2007). Parasitoids are important regulators of arthropod populations, including major agricultural pests (Godfray 1994). The genus *Aphelinus* comprises more than 90 recognized species (Hopper et al. 2012; Universal Chalcidoidea Database, www.nhm.ac.uk/entomology/chalcidoids/index.html, accessed 10 October 2016). Within *Aphelinus*, several complexes of closely related species provide excellent opportunities to explore genetic differentiation, speciation, and the evolution of reproductive compatibility, host use, and morphology (Heraty et al. 2007, Hopper et al. 2012). We studied species in three complexes of *Aphelinus*: (1) five species in the *Aphelinus varipes* (Förster,1841) complex from throughout Eurasia; (2) three species in the *Aphelinus mali* (Haldeman, 1851) complex from eastern Asia; (3) one species in the *Aphelinus daucicola* Kurdjumov, 1913 complex from North America. The *Aphelinus varipes* complex comprises 12 described species (Förster 1841, Hayat 1972, 1994, Hayat and Fatima 1992, Howard 1908, Kurdjumov 1913, Nikol’skaya and Yasnosh 1966, Pan 1992, Yasnosh 1963). The monophyly of the *Aphelinus varipes* complex is well supported by a combination of morphological and genetic characters (Heraty et al. 2007). However, some species within the complex show little morphological divergence, making identification difficult. The *Aphelinus mali* complex comprises 14 recognized species, some of which also show little morphological divergence (Ashmead 1888, Evans et al. 1995, Gahan 1924, Girault 1913, Haldeman 1851, Hayat 1998, Hopper et al. 2012, Prinsloo and Neser 1994, Timberlake 1924, Yasnosh 1963, Zehavi and Rosen 1988). The *Aphelinus daucicola* species complex comprises three species that differ from the members of the *Aphelinus mali* complex in several traits (Hopper et al. 2012). Using flow cytometry, we estimated the genome sizes of species in these complexes. We also made and examined chromosomal preparations to determine their karyotypes. We found consistent differences in genome size between complexes, and these differences correlated with differences in relative sizes estimated from karyotypes. We detected chromosomal rearrangements as well as karyotypic synapomorphies.

## Materials and methods

### Specimens

The parasitoid species studied, the sources of the colonies, and the permit and voucher numbers are listed in Table 1. These colonies were reared on aphids at the USDA-ARS, Beneficial Insect Introductions Research Unit, in Newark, Delaware, USA. Vouchers for these populations are maintained at -20 °C in 100% molecular grade ethanol at the Beneficial Insect Introduction Research Unit, Newark, Delaware. Females of the yellow-white strain of *Drosophila melanogaster* (Meigen, 1830) (stock number 1495, obtained from the Bloomington Drosophila Stock Center at Indiana University, http://flystocks.bio.indiana.edu) were used as internal controls for flow cytometry. All institutional and national guidelines for the care and use of laboratory animals were followed.

**Table 1.** The nine *Aphelinus* species studied, the year and country of their collection, permit and voucher numbers.

Species complex	Species	Authority	Year	Country	Permit and voucher
* Aphelinus varipes*	* Aphelinus atriplicis*	Kurdjumov, 1913	2000	Georgia	P526P-15-04274, VGg00_Dn
	* Aphelinus varipes*	(Förster, 1841)	2009	France	P526P-13-02503, VFr09_Rp
	* Aphelinus certus*	Yasnosh, 1963	2001	Japan	P526P-01-53096, VJp01_TU
	* Aphelinus kurdjumovi*	Mercet, 1930	2000	Georgia	P526P-13-02503, VGg00_Rp
	* Aphelinus hordei*	Kurdjumov, 1913	2011	France	P526P-15-04274, VFr11_Dn
* Aphelinus daucicola*	* Aphelinus daucicola*	Kurdjumov, 1913	2013	USA	P526P-15-04274, DUSA12_UD
* Aphelinus mali*	* Aphelinus glycinis*	Hopper et Woolley, 2012	2007	China	P526P-08-02142, MKor09_M
	* Aphelinus coreae*	Hopper et Woolley, 2012	2009	Korea	P526P-01-72318, MCh04_Bj
	* Aphelinus rhamni*	Hopper et Woolley, 2012	2005	China	P526P-01-53096, MCh05_Bj

### Flow cytometry

Live *Aphelinus* were sexed, flash frozen in liquid nitrogen, and stored at −80°C. To estimate genome sizes, we used the flow cytometry protocol described by Hanrahan and Johnston (2011) and Hare and Johnston (2011). We dissected heads from both males and females of the *Aphelinus* species in cold Galbraith buffer (Galbraith et al. 1983). Heads of female *Drosophila melanogaster* were used as internal standards (1C = 175 Mb or 0.17 pg). To release the nuclei from cells, heads from 15 female *Aphelinus* and one female *Drosophila* Fallén, 1823 for each replicate were ground together in one milliliter of cold Galbraith buffer using 15 strokes of the “A” pestle in a 2-ml Kontes Dounce tissue grinder. As with other Hymenoptera, *Aphelinus* species have haplodiploid sex determination, with males coming from unfertilized eggs and females from fertilized eggs. Thus males carried half as much DNA per cell as females, which made male genome sizes too close to that of *Drosophila melanogaster*. Thus when processed the heads of 15 males per replicate as described above, we included the heads from 15 females of the same parasitoid species as internal standards. The samples were passed through a 35 micron filter and then stained with 40 parts per million of propidium iodide in the dark for 3–5 hours at 4°C. Samples were analyzed with laser excitation at 488 nm on a Becton Dickinson FACSCalibur Flow Cytometer (BD Biosciences, San Jose, CA, USA) at CTCR Core Facility, University of Delaware. Red fluorescence from the propidium iodide was detected using an FL2 filter. Three to six replicates were measured for females and males of each species.

The haploid content of DNA in megabases (Mb) was calculated for each *Aphelinus* sample from the ratio of mean fluorescence of the sample to mean fluorescence of the standard times the genome size of the standard. We report genome size estimates in megabases, but also give estimates in picograms (pg) calculated by dividing the amount of DNA in Mb by the standard 1C value of 978 Mb.

### Karyotypes

Chromosome preparations were made from cerebral ganglia of prepupae using a modified version of the technique in Imai et al. (1988). Wasps were dissected in 0.5% hypotonic sodium citrate solution containing 0.005% colchicine, and the tissues were incubated in fresh solution for ~30 minutes at room temperature. The material was transferred to a pre-cleaned microscope slide using a Pasteur pipette and gently flushed with Fixative I (glacial acetic acid: absolute ethanol: distilled water 3:3:4). Tissues were disrupted in an additional drop of Fixative I using dissecting needles. Another drop of Fixative II (glacial acetic acid: absolute ethanol 1:1) was then applied to the center of the area and blotted off the edges of the slide. The slide was air dried for ~30 minutes at room temperature. For conventional staining, preparations were stained with freshly prepared 3% Giemsa solution in 0.05M Sørensen’s phosphate buffer (Na_2_HPO_4_ + KH_2_PO_4_, pH 6.8). Mitotic divisions were studied and photographed using an optic microscope Zeiss Axioskop 40 FL fitted with a digital camera AxioCam MRc (Carl Zeiss, Oberkochen, Germany). To obtain karyograms, the resulting images were processed with image analysis programs: Zeiss AxioVision version 3.1 and Adobe Photoshop version 8.0. Mitotic chromosomes were measured for 5–19 cells in 1–6 wasps per species using Adobe Photoshop. We report total length (μm) of all chromosomes in each karyotype for males and females; for diploid sets, we divided total length by two to make the values comparable to haploid sets. We also report relative lengths (RL: 100 × length of each chromosome divided by total length of the set) and centromeric indices (CI: 100 × length of shorter arm divided by total length of a chromosome) for females of each species. Chromosomes were classified into metacentric (M) or acrocentric (A) according to the guidelines in Levan et al. (1964).

### Fluorescence *in situ* hybridization

A custom biotinylated fragment from the 28S rDNA gene was used to probe *Aphelinus coreae* chromosomes with fluorescence *in situ* hybridization (FISH). To prepare the probe, we extracted DNA from ~50 adult parasitoids using a Qiagen DNeasy Blood and Tissue Kit (Qiagen, Valencia, CA, USA). From this DNA, we amplified a ~650 nt fragment of the 28S rDNA gene using the following primers and PCR protocol: reaction mix - 5 µl NEB PCR buffer and 0.5 µl *Taq* polymerase (New England Biolabs, Ipswich, MA, USA), 4 µl each of 2.5 mM dATP, dCTP, dGTP, 4 µl 0.25 mM dTTP plus 1 µl 1mM biotinylated-11-dUTP, 1 µl 10 µM forward primer (5’-cgt gtt gct tga tag tgc agc) and 1 µl 10 µM reverse primer (5’-tca aga cgg gtc ctg aaa gt), 4 µl genomic DNA (50 ng/µl), 21.5 µl ultrapure H_2_O; cycling - 3 min at 95 °C, then 35 cycles 95 °C for 30 sec, 55°C for 30 sec, and 72°C for 60 sec, and a final extension at 72 °C for 3 min. Unincorporated dNTPs, primers, and other unwanted components were removed from the PCR product using precipitation with sodium acetate and ethanol, and the resulting pellet was resuspended in 50 µl ultrapure H_2_O, yielding a solution of probe at 300 ng/µl.

Chromosomes were prepared for probing using the protocol described above for karyotyping. To probe the chromosomes, a protocol modified from Matsumoto et al. (2002) was used. Chromosomes were baked onto slides at 65°C and UV crosslinked in a Spectrolinker XL-1000 UV Crosslinker (Spectronics, Westbury, NY, USA) twice at 120 mJ/cm^2^. The slides were treated with RNase A, dehydrated with ethanol, denatured in formamide, and then dehydrated again with ethanol. The slides were then treated with proteinase K and dehydrated a third time with ethanol. Hybridization solution was prepared from the biotinylated probe (7 µg in 23 µl), formamide (90 µl), 30% dextran sulfate solution (60 µl), 10 mg/ml salmon sperm DNA (5 µl), and 20x SSC (22 µl). This solution was denatured at 95°C, placed on ice, and then 50 µl was applied to each slide, which were then covered with parafilm and left in a moist chamber at 37°C for 12–16 h. The slides were washed twice in formamide (50% in 2x SSC) and twice in 2x SSC with gentle shaking, and transferred to BN buffer (100 mM NaHCO_3_, 0.1% Nonidet P-40) for 10 min at room temperature. After this, blocking buffer (100 mM NaHCO_3_, 0.05% Nonidet P-40, 0.02% NaN_3_, 5% non-fat dry milk) was applied, and the slides were covered with parafilm and incubated for 10 min at room temperature. The buffer was removed, streptavidin-Alexa fluor 568 conjugate (Thermo Fisher Scientific, Waltham, MA, USA), diluted 1/50 in blocking buffer, was added, and the slides were covered with parafilm and incubated at 37°C for 1 h. The slides were washed with three changes of BN buffer in a light-tight chamber with gentle shaking. Signal enhancement was done following Pinkel et al. (1986). Fifty microliters of biotinylated goat anti-Avidin D (Vector Laboratories, Burlingame, CA, USA), diluted 1/50 in the blocking buffer, was applied to each slide, which were then covered with parafilm and incubated at 37°C for 1 hour. The slides were then washed with BN buffer, more streptavidin-labeled fluor was added, the slides were washed again with BN buffer, and then air-dried in the dark at room temperature. Anti-fade medium (ProLong Gold Antifade Reagent with DAPI, Cell Signaling Technology, Danvers, MA, USA) was added and a glass coverslip was placed over the chromosomal preparation. The chromosomes were imaged using a Zeiss 510 NLO Multiphoton microscope and a Zeiss Elyra PS 1 microscope (Carl Zeiss, Pleasanton, CA, USA) with confocal microscopy at the Bio-Imaging Center, Delaware Biotechnology Institute, Newark, DE, USA.

### Data analysis

Genome sizes and total lengths of chromosome sets were compared among species and between sexes in generalized linear models with species and sex as fixed main-effects and Poisson error distributions using the glm function in R (R Core Team 2014). The set of relative lengths among species in a multivariate analysis of variance was compared with the Pillai–Bartlett statistic and the manova function in R. Centromeric indexes among species in generalized linear models were compared for each chromosome with species as a fixed effect and Poisson error distributions using the glm function in R. Because chromosomal formulae were different for the *Aphelinus varipes* complex *versus* the *Aphelinus mali* and *Aphelinus daucicola* complexes, we analyzed the effects of species on relative lengths and centromeric indexes separately within these groups. For genome size, the experimental unit was either 15 heads of female parasitoids and one *Drosophila melanogaster* head pooled or 15 heads of male parasitoids and 15 heads of female parasitoids pooled. For total lengths of chromosome sets and relative lengths and centromeric indexes of chromosomes, the experimental unit was an individual mitotic cell. Post-hoc comparisons of means were done using the glht and cld functions in the multcomp package in R. We tested the relationships between genome sizes from flow cytometry and total lengths of chromosome sets with linear regression using the lm function in R. Data are archived on the Ag Data Commons website (data.nal.usda.gov; DOI 10.15482/USDA.ADC/1329930).

## Results

### Genome sizes from flow cytometry

Haploid genome sizes of *Aphelinus* differed among species (model deviance = 444.0; residual deviance = 5.4; df = 6, 60; *P* < 0.0001). Female genome sizes ranged from 330 to 483 Mb so the largest was 1.5 times the smallest (Table 2, which also shows results of multiple comparisons among means of each species). Female and male *Aphelinus* had similar haploid genome sizes, with female and male sizes within 2–13 Mb (1–4 percent) of one another, so the sexes did not differ significantly (model deviance = 1.1; residual deviance = 4.3; df = 1, 59; *P* = 0.30). Genomes (averaged across sexes) in the *Aphelinus mali* complex were significantly larger (37–148 Mb or 9–44 percent) than those *Aphelinus varipes* complex, and genomes in the *Aphelinus varipes* complex were significantly larger (1–59 Mb or 1–18 percent) than those in the *Aphelinus daucicola* complex (model deviance = 378.10; residual deviance = 73.3; df = 6, 60; *P* < 0.0001). The genome of *Aphelinus rhamni* was significantly larger (43–53 Mb or 10–12 percent) than the genomes of the other species in the *Aphelinus mali* complex. The genome of *Aphelinus hordei* was significantly larger (40–58 Mb or 9–17 percent) than the genomes of the other species in the *Aphelinus varipes* complex.

**Table 2.** Haploid genome sizes of nine *Aphelinus* species estimated from flow cytometry. Shared letters after means indicates that they do not differ significantly.

Species complex	Species	Sex	n replicates	Genome size		95% CI
				(pg)	(Mb)	(Mb)
* Aphelinus varipes*	* Aphelinus atriplicis*	female	6	0.361	353a	338–368
		male	3	0.366	358a	337–380
	* Aphelinus varipes*	female	3	0.340	333a	313–354
		male	3	0.348	340a	320–362
	* Aphelinus certus*	female	6	0.369	361a	347–377
		male	3	0.375	367a	346–390
	* Aphelinus kurdjumovi*	female	4	0.356	348a	331–367
		male	3	0.363	355a	334–377
	* Aphelinus hordei*	female	4	0.402	393b	374–412
		male	3	0.406	397b	375–421
* Aphelinus daucicola*	* Aphelinus daucicola*	female	4	0.337	330a	313–348
		male	3	0.351	343a	322–364
* Aphelinus mali*	* Aphelinus glycinis*	female	6	0.442	432c	416–449
		male	5	0.441	431c	413–450
	* Aphelinus coreae*	female	3	0.449	439c	416–464
		male	3	0.454	444c	421–468
	* Aphelinus rhamni*	female	6	0.494	483d	466–501
		male	3	0.498	487d	463–513

### Karyotypes

**Figure 1.**
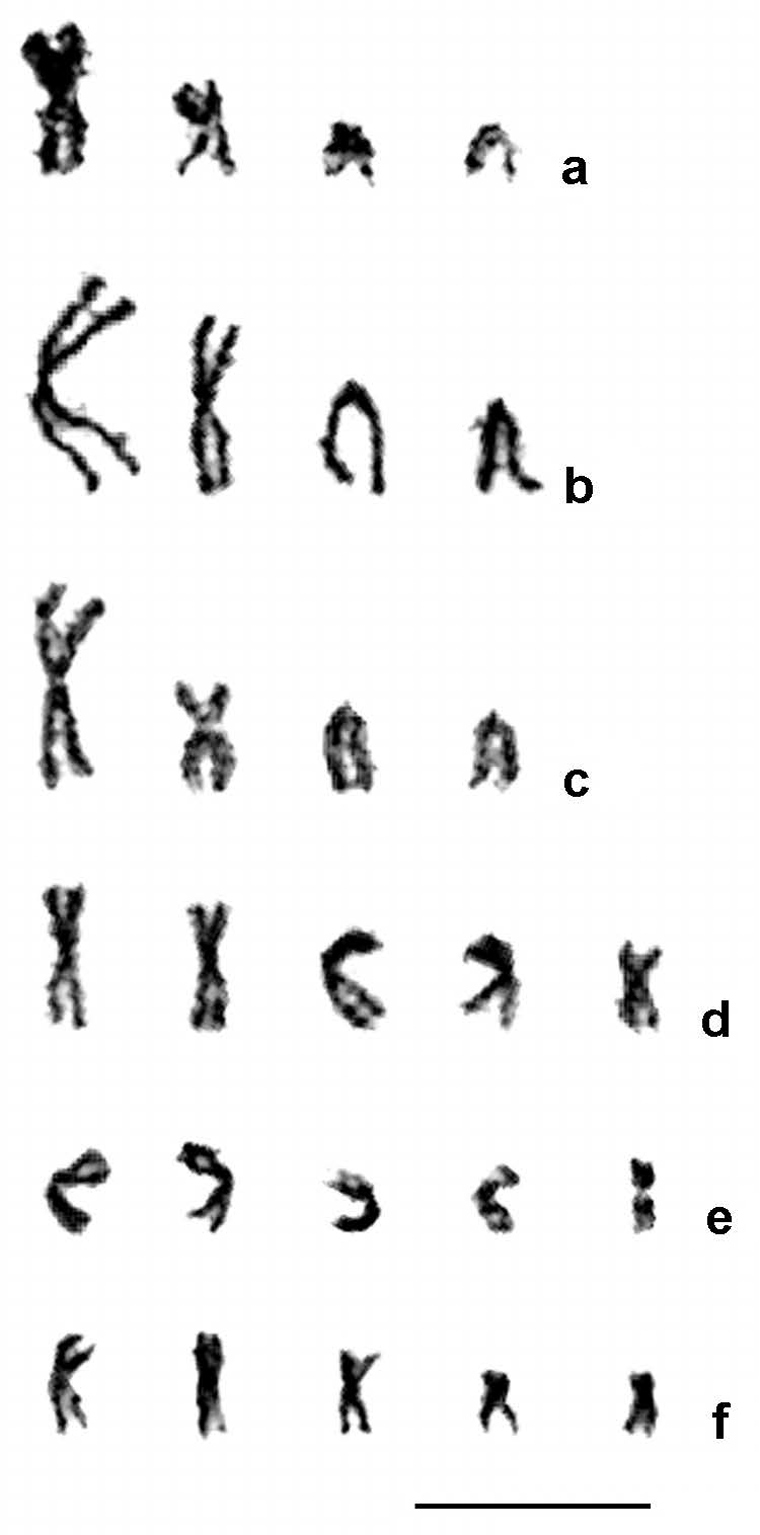
Haploid mitotic karyograms of six *Aphelinus* species. **a** *Aphelinus atriplicis* **b** *Aphelinus certus* **c** *Aphelinus hordei* **d** *Aphelinus coreae* **e** *Aphelinus rhamni* **f** *Aphelinus daucicola*. Species in the *Aphelinus varipes* complex have n = 4 *versus* n = 5 in the *Aphelinus mali* and *Aphelinus daucicola* complexes. Scale bar: 10 µm.

**Figure 2.**
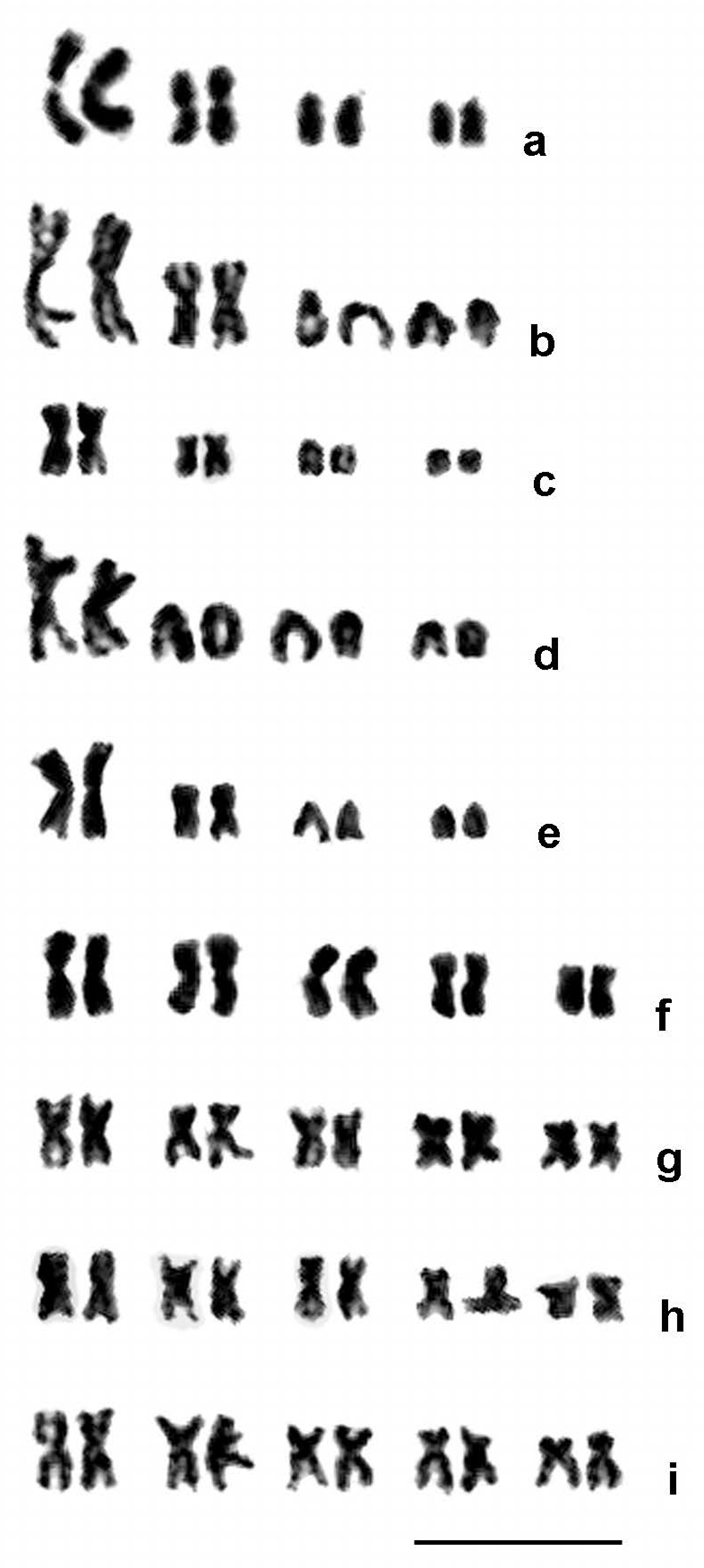
Diploid mitotic karyograms of nine *Aphelinus* species. **a** *Aphelinus atriplicis* **b** *Aphelinus certus* **c** *Aphelinus hordei* **d** *Aphelinus kurdjumovi* **e** *Aphelinus varipes* **f** *Aphelinus coreae* **g** *Aphelinus glycinis* **h** *Aphelinus rhamni*, **i** *Aphelinus daucicola*. Species in the *Aphelinus varipes* complex have 2n = 8 *versus* 2n = 10 in the *Aphelinus mali* and *Aphelinus daucicola* complexes. Scale bar: 10 µm.

Species in the *Aphelinus varipes* complex had four chromosomes in haploid males and thus eight chromosomes in diploid females, whereas species in the *Aphelinus mali* and *Aphelinus daucicola* complexes had five chromosomes in haploids and thus ten chromosomes in diploids (Table 3; Figs 1–2). Karyotypes of the *Aphelinus varipes* complex usually included a large metacentric chromosome 1 and a small metacentric chromosome 2 and small acrocentric chromosomes 3 and 4, except for *Aphelinus kurdjumovi*, in which the small metacentric chromosome 2 appears to have been replaced by an acrocentric chromosome 2 of similar size; whereas species in the *Aphelinus mali* and *Aphelinus daucicola* complexes had metacentric chromosomes only, and their chromosomes showed a continuous gradation in length (Tables 4 and 5). Relative lengths of chromosome sets differed significantly among species in the *Aphelinus varipes* complex (*F* = 3.2; df = 16, 540; *P* <0.0001), but did not quite differ significantly among species in the *Aphelinus mali* and *Aphelinus daucicola* complexes (*F* = 1.7; df = 12, 255; *P* = 0.07).

**Table 3.** Karyotypic features of nine *Aphelinus* species. Shared letters after means indicate that they do not differ significantly within each sex.

	species complex	species	number nuclei measured	number chromosomes	chromosomal formula	total length of chromosome set (µm)	
						mean	95% confidence interval
female	* Aphelinus varipes*	* Aphelinus atriplicis*	14	8	4M + 4A	15.6ac	13.7–17.9
		* Aphelinus varipes*	16	8	4M + 4A	15.1ab	13.3–17.1
		* Aphelinus certus*	19	8	4M + 4A	14.0a	12.4–15.8
		* Aphelinus kurdjumovi*	13	8	2M + 6A	16.8ac	14.8–19.2
		* Aphelinus hordei*	8	8	4M + 4A	14.3ab	11.9–17.1
	* Aphelinus daucicola*	* Aphelinus daucicola*	15	10	10M	16.8ac	14.8–19.0
	* Aphelinus mali*	* Aphelinus glycinis*	5	10	10M	19.6ac	16.1–23.9
		* Aphelinus coreae*	6	10	10M	21.3c	17.9–25.4
		* Aphelinus rhamni*	19	10	10M	18.4bc	16.5–20.4
male	* Aphelinus varipes*	* Aphelinus atriplicis*	3	4	2M + 2A	16.3ab	12.3–21.6
		* Aphelinus certus*	3	4	2M + 2A	25.0bc	19.9–31.3
		* Aphelinus hordei*	21	4	2M + 2A	17.8b	16.1–19.7
	* Aphelinus daucicola*	* Aphelinus daucicola*	7	5	5M	23.7ac	20.4–27.6
	* Aphelinus mali*	* Aphelinus coreae*	6	5	5M	29.8c	25.8–34.5
		* Aphelinus rhamni*	4	5	5M	22.5bc	18.3–27.7

M = metacentric; A = acrocentric.

**Table 4.** Relative lengths of chromosomes in *Aphelinus* species. Means with 95% confidence intervals in parentheses.

**Species complex**	**Species**	**Chromosome**							
		**1**	**2**		**3**		**4**		**5**
		**Relative length**							
* Aphelinus varipes*	* Aphelinus atriplicis*	40		26		18		16	
		(38–42)		(24–28)		(17–20)		(14–17)	
	* Aphelinus varipes*	41		26		18		15	
		(39–43)		(24–27)		(17–19)		(14–17)	
	* Aphelinus certus*	40		26		18		16	
		(38–43)		(24–28)		(17–20)		(14–17)	
	* Aphelinus kurdjumovi*	43		24		18		15	
		(40–45)		(22–26)		(17–20)		(14–17)	
	* Aphelinus hordei*	41		25		19		16	
		(37–44)		(23–27)		(17–21)		(14–18)	
* Aphelinus daucicola*	* Aphelinus daucicola*	24		22		19		18	18
		(23–26)		(20–24)		(18–21)		(17–20)	(17–20)
* Aphelinus mali*	* Aphelinus glycinis*	24		22		21		18	18
		(21–27)		(19–25)		(18–24)		(16–21)	(16–21)
	* Aphelinus coreae*	24		22		20		18	18
		(21–27)		(20–25)		(18–23)		(16–21)	(16–21)
	* Aphelinus rhamni*	24		22		20		18	18
		(23–26)		(21–24)		(19–22)		(17–19)	(17–19)

**Table 5.** Centromeric indexes of chromosomes in *Aphelinus* species. Means (95% confidence intervals); shared letters within a species complex and chromosome indicate means that are not significantly different.

**complex**	**species**	**Chromosome**				
		**1**	**2**	**3**	**4**	**5**
		**Centromeric index**				
* Aphelinus varipes*	* Aphelinus atriplicis*	46a	47b	0	0	
		(44–49)	(45–50)			
	* Aphelinus varipes*	46a	47b	0	0	
		(44–49)	(45–49)			
	* Aphelinus certus*	46a	46ab	0	0	
		(44–49)	(44–48)			
	* Aphelinus kurdjumovi*	47a	0	0	0	
		(44–49)				
	* Aphelinus hordei*	47a	41a	0	0	
		(44–51)	(38–45)			
* Aphelinus mali* and *Aphelinus daucicola*	* Aphelinus daucicola*	45a	45a	41a	39a	38a
		(43–48)	(43–48)	(38–43)	(37–41)	(36–40)
	* Aphelinus glycinis*	48a	44a	44ab	44ab	42ab
		(43–52)	(40–48)	(40–48)	(40–49)	(39–47)
	* Aphelinus coreae*	44a	47a	43ab	44ab	44b
		(41–48)	(43–51)	(40–47)	(40–47)	(40–47)
	* Aphelinus rhamni*	46a	46a	45b	45b	43b
		(44–49)	(44–48)	(43–47)	(43–47)	(41–45)

**Figure 3.**
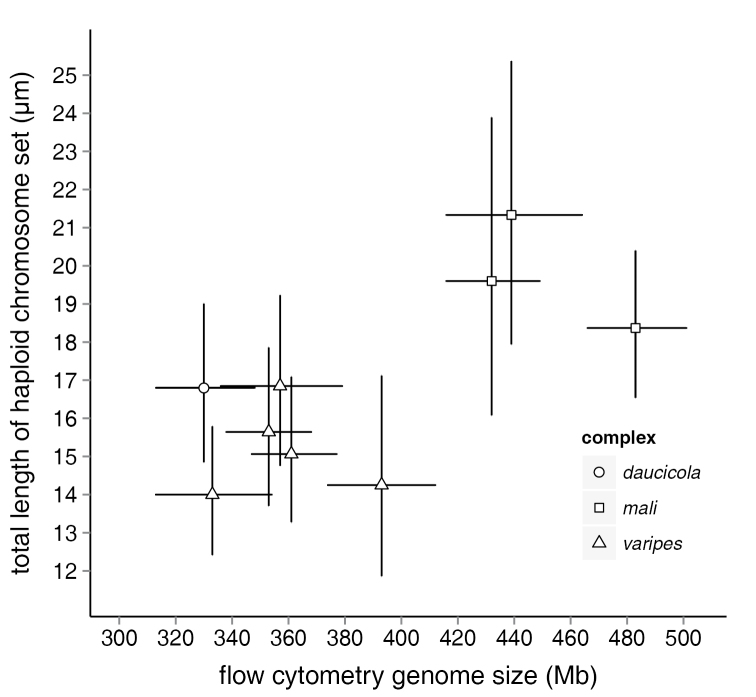
Total length of chromosome set (µm) *versus* flow cytometry genome size (Mb) for nine *Aphelinus* species. Error bars are 95% confidence intervals.

Centromeric indexes for chromosome 1 did not differ among species, and centromeric indexes for chromosome 2 did not differ among species in the *Aphelinus mali* and *Aphelinus daucicola* complexes. However, in the *Aphelinus varipes* complex, the centromeric index of chromosome 2 in *Aphelinus hordei* was significantly lower than in other members of the *Aphelinus varipes* complex. Centromeric indexes for chromosomes 3 and 4 in *Aphelinus daucicola* were significantly lower than those for *Aphelinus rhamni*, and the centromeric index for chromosome 5 in *Aphelinus daucicola* was significantly lower than those for *Aphelinus coreae* and *Aphelinus rhamni* (Table 6). Total lengths of chromosome sets differed among species (model deviance = 65.1; residual deviance = 153.2; df = 8, 150; *P* < 0.0001) and between sexes (model deviance = 34.3; residual deviance = 118.9; df = 1, 149; *P* < 0.0001). Total lengths ranged from 14 to 21 µm so the longest set was 1.5 times the shortest (Table 3). Total lengths were significantly greater in the *Aphelinus mali* complex than in the *Aphelinus varipes* complex for both males and females, with the values in *Aphelinus daucicola* complex intermediate between these extremes (females: model deviance = 20.5; residual deviance = 92.8; df = 2, 112; < 0.0001; males: model deviance = 26.2; residual deviance = 38.8; df = 2, 41; *P* < 0.0001). Mean total chromosome length correlated with mean genome size estimated from flow cytometry (*F* = 6.3; df = 1, 7; *P* = 0.04; *r*^2^ = 0.47; Fig. 3), primarily because of the difference in total chromosome length between the *Aphelinus mali* and *Aphelinus varipes* complexes.

**Table 6.** Analysis of deviance for differences in centromeric indexes among species of *Aphelinus*; acrocentric chromosomes were not included in these analyses.

**complex**	**chromosome**	**model**		**residual**		
		**df**	**deviance**	**df**	**deviance**	**P**
*A varipes*	1	4	0.2	135	23.1	1.00
	2	3	9.3	110	22.3	0.03
* Aphelinus mali* and *Aphelinus daucicola*	1	3	1.8	86	20.7	0.62
	2	3	1.2	86	23.0	0.74
	3	3	7.2	86	33.2	0.07
	4	3	16.6	86	29.0	0.0008
	5	3	12.7	86	52.8	0.005

**Figure 4.**
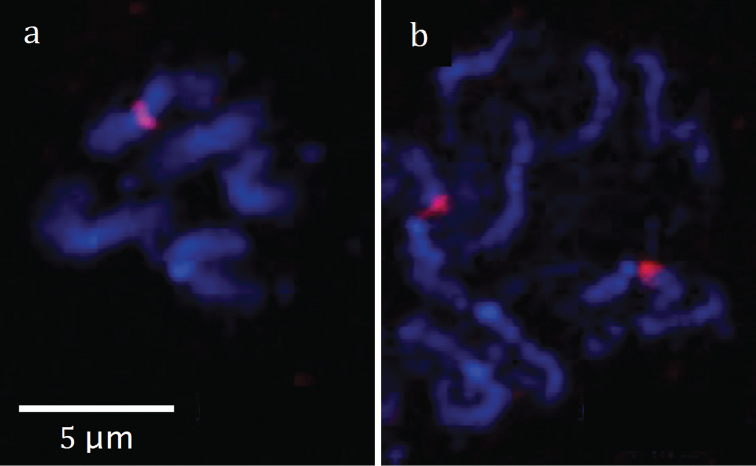
Fluorescence *in situ* hybridization with 28S rDNA probe. **a** metaphase chromosomes of the haploid karyotype and **b** prometaphase chromosomes of the diploid karyotype of *Aphelinus coreae*. Red = hybridization signal (a single rDNA cluster in the haploid set and paired clusters in the diploid set), blue = counterstaining of chromosomes with DAPI.

Hybridization with a 28S rDNA probe revealed a single rDNA cluster on chromosomes of the haploid set and two rDNA clusters in the diploid set (Fig. 4). These clusters were near the centromere on a medium-sized metacentric chromosome.

## Discussion

The large genome size differences between the *Aphelinus varipes* complex *versus* the *Aphelinus mali* complex matched the phylogenetic divergence between these complexes (Heraty et al. 2007). The difference between the complexes is also supported by the difference in karyotypes: four chromosomes with one-two metacentrics and two-three acrocentrics in the *Aphelinus varipes* complex *versus* five metacentric chromosomes in the *Aphelinus mali* complex. However, the additional chromosome in the *Aphelinus mali* complex does not necessarily account for a corresponding increase in genome size, because *Aphelinus daucicola* also has five chromosomes and yet has the smallest genome we observed. Four haploid genome sizes have been published for the family Aphelinidae, including an estimate of 635 Mb (=0.65 pg) for *Aphelinus abdominalis* (Dalman, 1820) (Ardila-Garcia et al. 2010) which is much larger than our estimates for other species of *Aphelinus*. However, beyond *Aphelinus*, other Hymenoptera genera have genome sizes spanning wide ranges (e.g., some ants, *Tapinoma* Förster, 1850, 362–597 Mb; *Solenopsis* Westwood, 1840, 372–753 Mb; http://www.genomesize.com). This also applies to some genera of parasitoids, e.g. *Leptopilina* Förster, 1869 (Figitidae) with genome sizes ranging from 363 to 520 Mb (Gokhman et al. 2011).

Genome size has been hypothesized to depend on several factors, including eusociality, parasitism, and developmental biology in insects (Ardila-Garcia and Gregory 2009, Gregory 2002, Johnston et al. 2004). These hypotheses come from the associations between genome size, cell size, and cell division rates found in many taxa (Ardila-Garcia and Gregory 2009, Gregory 2005). The mean genome size for species of parasitic Hymenoptera (293 Mb) does not differ greatly from the mean genome size for species of eusocial Hymenoptera (333 Mb), but the genomes for both groups are significantly smaller than those for species of non-parasitoid solitary Hymenoptera (469 Mb) (Ardila-Garcia and Gregory 2009). However, it is unclear why there should be so much variation in genome size among species of *Leptopilina* or *Aphelinus*, given the very similar biologies within each genus.

**Figure 5.**
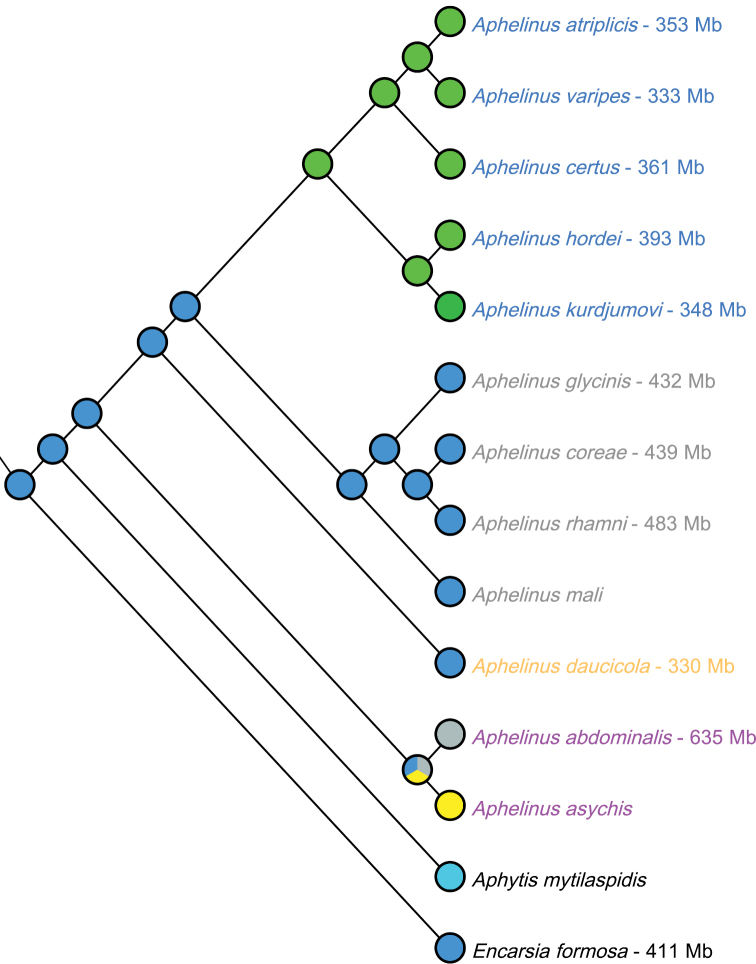
Chromosomal formulae and genome sizes on phylogeny of *Aphelinus* species and several outgroups. Blue = 5 metacentric chromosomes; aqua = 1 metacentric and 4 acrocentric chromosomes; green = 2 metacentric and 2 acrocentric chromosomes; dark green = 1 metacentric and 3 acrocentric chromosomes; yellow = 2 metacentric, 1 subtelocentric, and 1 acrocentric chromosome; grey = unknown. Numbers after species names are genome sizes estimated from flow cytometry; values for *Aphelinus abdominalis* and *Encarsia formosa* are from (Ardila-Garcia et al. 2010). The different colors for species of *Aphelinus* indicate membership in the four species complexes for which data are available.

Mapping karyotypic data on a molecular phylogeny of *Aphelinus* and two outgroup species allowed reconstruction of karyotype evolution in the species we studied (Fig. 5). The phylogeny was modified from Heraty et al. (2007) with results from Heraty et al. (2013), Kim and Heraty (2012), and unpublished data. Chromosomal formulae were mapped on the phylogeny using Mesquite (Maddison and Maddison 2016). Chromosomal formulae for *Aphytis mytilaspidis* (Le Baron, 1870) and *Encarsia formosa* Gahan, 1924 are from (Gokhman 2003). Concerning chromosome number, *Aphelinus asychis* Walker, 1839, has four chromosomes (Gokhman 2003), which is what we found for all the species we studied in the *Aphelinus varipes* complex. On the other hand, we found five chromosomes for *Aphelinus daucicola* and three species in the *Aphelinus mali* complex, which is also the chromosome number reported for *Aphelinus mali* (Haldeman, 1851) (Viggiani 1967). The *Aphelinus varipes* and *Aphelinus mali* complexes are sister clades, but *Aphelinus asychis* is the most basal species in the phylogeny of the genus, at least for the species for which there are phylogenetic data. This information alone would suggest four chromosomes is the ancestral state. However, five or six chromosomes are the numbers most frequently reported in chalcidoids (although it has been hypothesized that 9–10 is the ancestral state) (Gokhman 2009). Furthermore, five chromosomes has been reported for *Aphytis mytilaspidis* (Rössler and DeBach 1973), a species in the genus most closely related to *Aphelinus* for which chromosome number has been reported, and five chromosomes have been reported for many species of *Encarsia* Förster, 1878, a genus of aphelinids in another subfamily of Aphelinidae (Gokhman 2009). Chromosomal fusion (and hence decreased chromosome number) is a trend of karyotype evolution in many groups of organisms, including parasitic Hymenoptera (Gokhman 2009). Chromosomal fissions are also possible, but they are substantially less frequent, probably because they break existing linkage groups and therefore can decrease fitness. Thus, the reduced chromosome number in *Aphelinus asychis* has probably arisen independently of that in the *Aphelinus varipes* complex, because these two groups also have different karyotype structures. *Aphelinus asychis* has a haploid karyotype with two metacentric chromosomes, a subtelocentric chromosome and an acrocentric chromosome, with the three smallest chromosomes being similar in size (Gokhman 2009). The same chromosome number in *Aphelinus asychis* and in the *Aphelinus varipes* complex could be an example of karyotypic orthoselection (White 1973), i.e. similar karyotypes with independent origins. However, the hypothesis of chromosomal fusion giving rise to four chromosomes in the *Aphelinus varipes* complex cannot explain the significant and substantially smaller genome sizes in this complex compared to those in the *Aphelinus mali* complex.

Chromosomes in the *Aphelinus varipes* complex differ from those in the *Aphelinus mali* complex in relative length and centromeric indices. The longest metacentric chromosome in species in the *Aphelinus varipes* complex is much longer than the other chromosomes. We suggest that this metacentric chromosome resulted from a fusion of two smaller chromosomes from an ancestral karyotype with five chromosomes. Species in the *Aphelinus varipes* complex have two smaller acrocentrics that, in turn, could originate from metacentric chromosomes of the ancestral karyotype via pericentric inversions. Moreover, the position of the centromere of the second largest chromosome underwent further changes in two sister species in the *Aphelinus varipes* complex, *Aphelinus kurdjumovi* and *Aphelinus hordei*. The centromere is significantly shifted in *Aphelinus hordei* (CI= 41 *versus* 46 in *Aphelinus certus*, and 47 in *Aphelinus atriplicis* and *Aphelinus varipes*), and is further moved to a terminal position in *Aphelinus kurdjumovi* (CI= 0). We propose that consecutive pericentric inversions in *Aphelinus hordei* and *Aphelinus kurdjumovi* would be the most parsimonious explanation. These chromosomal rearrangements in the *Aphelinus varipes* complex are an example of a general trend in karyotype evolution in parasitic Hymenoptera, namely, karyotypic dissymmetrization, which involves an increase in size differentiation between chromosomes and an increase in the proportion of acrocentric chromosomes (Gokhman 2009).

A recent review of the distribution of rDNA sites on chromosomes of parasitic Hymenoptera showed that the number of these sites correlates with chromosome number (Gokhman et al. 2014). We found that this is also true for at least one *Aphelinus* species: haploid males of *Aphelinus coreae*, with their low number of chromosomes (*n* = 5), had a single rDNA site, while diploid females of *Aphelinus coreae* had two rDNA sites. Fluorescence *in situ* hybridization is especially useful for studying karyotypes with morphologically similar chromosomes that are difficult to recognize with conventional staining, like the chromosomes of *Aphelinus coreae* and other species in the *Aphelinus mali* and *Aphelinus daucicola* complexes.

Total chromosomal length was correlated with genome size in *Aphelinus*, but this was because of the difference in chromosome length between the *Aphelinus mali* and *Aphelinus varipes* complexes. Although a similar correlation was found for species in the family Figitidae (Gokhman et al. 2014), only large differences in chromosome length were distinguished in both cases, probably because of intraspecific variation in chromosomal condensation. Total lengths of male chromosomes exceeded those of females, although male and female genome sizes did not differ, and indeed it would be surprising if they did, given that males inherit their chromosomes from their mothers. The difference in the chromosome length between males and females may have resulted from differences in chromosomal condensation between the sexes, and this could compensate for the differences in chromatin available for transcription in the haploid and diploid genomes.

## Conclusions

Differences as large as 44% were found in genome size between *Aphelinus* species, which is surprising given the similarity in their morphology and life history. Mean total chromosome length correlated with mean genome size. The differences in genome size and total chromosome length between species complexes matched the phylogenetic divergence between species complexes. Chromosomal rearrangements in the *Aphelinus varipes* complex are an example of karyotypic dissymmetrization, which involves an increase in size differentiation between chromosomes and an increase in the proportion of acrocentric chromosomes, which is a general trend in karyotype evolution in parasitic Hymenoptera.
